# Intersections of Forced Migration and Gender in Physical Education

**DOI:** 10.3389/fsoc.2020.539020

**Published:** 2020-10-14

**Authors:** Fabienne Bartsch, Bettina Rulofs

**Affiliations:** ^1^Institute of Sociology and Gender Studies, German Sport University Cologne, Cologne, Germany; ^2^Department of Sports Sociology, Institute of Sport Science, University of Wuppertal, Wuppertal, Germany

**Keywords:** forced migration, gender, intersections, interviews, physical education, stereotypes, students from refugee backgrounds

## Abstract

Rising refugee movements have affected global society in general and the social system of sports in particular. The main structures and discourses of the sports system are also reflected in physical education (PE) at schools, which can, therefore, be regarded as an integral part of it. PE can play a significant role in the sports biographies of young people from refugee backgrounds. It is the only organizational framework of sports that all children and adolescents experience, and for many young people from refugee backgrounds it is the first and sometimes the only context in which they come into contact with sports in the host country. Consequently, PE teachers have a special opportunity to support students from refugee backgrounds in discovering their sports-related interests and to encourage them to deepen these interests outside school. Research suggests that teachers interact with learners from refugee backgrounds with stereotypical expectations for how they should behave and act. These expectations are structured along gender-specific logics, with male persons from refugee backgrounds often being classified as violent and threatening and female individuals from refugee backgrounds as fragile and oppressed. Against this background, through an interview study with 31 PE teachers from schools in Germany, we investigate teachers' perspectives on young people from refugee backgrounds. Specifically, we examine which intertwined gendered and racialized patterns of perception and interpretation become apparent when PE teachers talk about this group of students. Constructivist, intersectional, and postcolonial approaches are used to analyze the interviews and answer the research questions. The analytical screening of the interviews reveals that gendered and racialized perceptions of threat and vulnerability dominate the teachers' mindsets. Four main patterns that follow these gendered and racialized thoughts can be detected in the data material: *victimization and vulnerabilization, notions of threat and impulsivity, claims for assimilation and normalization*, and *demands for discipline*. These perceptions can influence the developmental opportunities of students from refugee backgrounds and reduce their participation and involvement in school sports and out-of-school sports contexts. Based on these results, we discuss strategies that might support (future) teachers in breaking stereotypes and overcoming narrow perceptions of young people from refugee backgrounds.

## Introduction

Wars, human rights violations, environmental changes, and other developments are forcing an increasing number of people to leave their homes. At the end of 2019, the world's forcibly displaced population reached a new record of 79.5 million (UNHCR, 2020). The vast majority of displaced individuals seek protection in their home countries or in neighboring states. However, the situation there is often similarly precarious, leading many to take the arduous route to more distant destinations, such as Europe. Within Europe, Germany is among the 10 countries with the highest number of first-time asylum applicants per million inhabitants (Eurostat, 2020), and worldwide, Germany is currently the country with the fifth largest absolute number of refugees (1.1 million) (UNHCR, 2020). Among those seeking protection in Germany are numerous children and young people. In 2019, every second asylum application of the total of 142,509 applications in Germany was filed for minors (Federal Office for Migration and Refugees, 2020). The considerable number of young people from refugee backgrounds has had an impact on German society in general and the social system of sports in particular, which is believed to play a crucial role in orienting new arrivals in their new environment by providing opportunities for confidence building, language acquisition, and the development of social relationships (Spaaij, 2015; Block and Gibbs, 2017). The main social structures and collective discourses of the sports system (e.g., physical nature, competition orientation, and potential for integration) are also reflected in physical education (PE) in schools—the first and sometimes the only organizational framework of sports with which children in general and young people from refugee backgrounds in particular come into contact. Thus, PE plays a significant role in socialization processes into sports and the formation of the sports biographies of young people. However, PE is not only a mirror of the out-of-school sports system but also a bridge and stepping stone into it, if properly managed. PE introduces young people to a wide range of physical activities, and it is these experiences that shape and encourage participation and involvement in out-of-school sports (Polet et al., 2019). On the other hand, negative experiences in PE may discourage young people from refugee backgrounds from becoming involved in other areas of sports outside school—for example, as active club members or in club management. PE, therefore, has a key and guiding function in the sports-related socialization of young learners from refugee backgrounds and has an impact on their further sporting careers outside school. For those without a migrant background, PE can be a formative field for the perception of the status of persons from refugee backgrounds in sports. In other words, school sports can be a significant and formative force for the positioning of people from refugee backgrounds in sports as well as for their perception and recognition in sports-related society.

Consequently, PE teachers have a special opportunity to support students from refugee backgrounds in discovering their sport-related interests and to encourage them to deepen these interests beyond school, in line with PE curricula that formulate the goal of lifelong physical activity and health (Conference of Ministers of Education Cultural Affairs, 2005). However, this can only succeed if PE teachers, who are usually part of the majority culture and thus occupy privileged social positions (Anttila et al., 2018), meet students from refugee backgrounds without prejudice and give them the opportunity to develop freely. Such an unbiased encounter can be hindered by the likelihood that many students from refugee backgrounds have little experience with sports, are not familiar with Western sports culture, or have not received any education in sports in their home country (Doherty and Taylor, 2007). PE teachers in Germany, on the other hand, have largely been socialized in performance-oriented and comparative competitive sports and have developed specific expectations and approaches to sports (Klinge, 2003). They can thus be understood as representatives of the organized sports system, who have internalized and constantly (re-)produce the prevailing discourses of this world (e.g., performance and success orientation, competition) and Western body and clothing ideals (body and muscle intonation, permissive clothing style). Furthermore, research emphasizes that PE should be seen as reflecting a society's general social and political climate (Macdonald et al., 2009). As in other countries, racist attitudes and prejudices against foreigners have become part of mainstream political discourse in Germany and contribute to increased support for anti-immigrant parties and extreme right-wing organizations [e.g., AfD [Alternative for Germany], PEGIDA [Patriotic Europeans Against the Islamization of the West]], which affects all areas of society, including the sports and school systems. Studies show that even PE teachers cannot absolve themselves from stereotypes about their students that are linked to nationality, culture, or religion (e.g., Dagkas et al., 2011; Benn and Pfister, 2013; Flintoff and Dowling, 2019). However, there have been only a few studies that have collected empirical data on students from refugee backgrounds in PE, who have often been confronted with extreme experiences and live in precarious circumstances, which makes them particularly vulnerable to stereotypical and biased expectations (Hynie, 2018).

Research suggests that stereotypes and expectations toward people from refugee backgrounds develop on the basis of gender-specific logics that frame Europe as an advanced region in gender equality and mock non-Western cultures as inherently misogynistic and therefore reactionary (Gray and Franck, 2019). The observation that people from refugee backgrounds are divided along the binary gender order into “‘bad’ and ‘good’ refugees” also influences this picture (Szczepanik, 2016, p. 29). A “good refugee” is associated with mainly “female” connotations: passive, needing, suffering, fragile, oppressed, and willing to adapt and subordinate. Male persons from refugee backgrounds who have repeatedly been portrayed in the media as a threatening, barbaric collective, do not correspond to this concept and are therefore widely regarded as “bad refugees” (Szczepanik, 2016). Moreover, the perception of male persons from refugee backgrounds in Germany is strongly shaped by the events of the Cologne New Year's Eve 2015/2016, when numerous women were exposed to massive sexual harassment by “non-German” men in the area of the central station in Cologne. Since this incident, images of male persons from refugee backgrounds as culturally different and even as potential aggressors, as well as retrogressive misogynist oppressors, have become much more accepted and commonplace. This has contributed to the development in which intertwined “gendered and racialized images of threat and vulnerability through which refugees come to be read *as* [a] risk or as *at* risk” came to dominate Western concepts of people from refugee backgrounds (Gray and Franck, 2019, p. 278).

Against this background, we examine in this article how teachers perceive and construct students from refugee backgrounds in PE. More specifically, we focus on gendered and racialized patterns of perception and interpretation that emerge when PE teachers talk about this group of students. In order to address these research issues, qualitative data from an interview study with 31 PE teachers from Germany is analyzed against the backdrop of constructivist, intersectional, and postcolonial approaches, which aim to scrutinize asymmetric power relations and break stereotypical representations of the (colonial) “other.” In general, the study aims to contribute to the small but existing knowledge base about students from refugee backgrounds and the intersections of forced migration and gender in PE by providing empirical evidence. Based on the empirical results, we discuss strategies that might support (future) PE teachers in overcoming migration- and gender-related stereotypes that reduce the developmental opportunities of male and female learners from refugee backgrounds in (school) sports.

## Literature Background and Theory

### Clarifying Relevant Terms

There is considerable scientific discourse about the classification of individuals on the criteria of race and gender that cannot be fully captured in this article. Both concepts have grown historically and are considered sensitive and complex. Many scientists agree that the idea of race originated mainly in the eighteenth and nineteenth centuries, when it was used to distinguish human populations on the basis of shared biological traits and external appearance, such as skin color, body shape, and hair types (Møller and Møller, 2017). Aside from this biological approach, race can also be regarded from a constructivist perspective, which we pursue in this article. From this point of view, race is understood as “a social construction that has aided in the perpetuation of hegemonic ideologies that promote racism and race-based discrimination” (Carter-Francique and Flowers, 2013, p. 74). This includes, for example, the belief that lighter skin tones reflect a higher degree of intelligence and civility (Sutherland, 2017). The term race is mainly used in the Anglo-Saxon world, whereas in Germany, it is often avoided for historical reasons closely related to the racial ideology of the National Socialists. Instead, the term “persons with migrant background” is employed. In population statistics, this term is usually understood as an objective and statistically unambiguous category that includes the following group of people:

“all foreigners and naturalized foreigners, all individuals who immigrated as ethnic Germans to the territory of today's Federal Republic of Germany after 1949, and all German citizens born in Germany with at least one parent who immigrated to Germany or was born in Germany as a foreign citizen” (Braun and Nobis, 2017, p. 186).

However, from the perspective of other approaches (e.g., social constructivism) on which this paper is based, this category is regarded as “a specifically German variant of the general sociological construct of foreignness, which describes a condition of perceived difference between groups defined by cultural, geographical, biological, and/or linguistic criteria” (Salentin, 2014, p. 26). This article focuses on a particular subgroup of persons with migrant background—people from refugee backgrounds, who tend to live in more precarious circumstances than other migrants. Many individuals from refugee backgrounds have been confronted with stressful and traumatic experiences (Schroeder et al., 2018). These trauma-related burdens and other circumstances, such as language barriers, uncertain future prospects, low economic resources, and limited social networks, make them particularly vulnerable to stereotypes and prejudices (Hynie, 2018). Furthermore, people from refugee backgrounds experience more restrictive policies in their new country that limit their access to social services, housing, and education (Bloch and Schuster, 2002), which may influence their chances of participation in sports and in society in general. However, we understand forced migration “not only as a phenomenon involving movements of people across borders, but also as a phenomenon of discourses, and to that extent as a phenomenon of hegemonic power relations” (Mecheril, 2018, p. 121). Based on this, we aim to analyze hegemonic power relations in PE teachers' discourses about students from refugee backgrounds and whether they are interwoven with gendered power relations.

Similar to race, gender was initially considered a biological category of analysis. In the course of time, the focus has shifted, and in social sciences, gender is now primarily understood as a social category that is built upon social norms, interwoven, and permeated by power relations (Sutherland, 2017). In this sense, we refer to gender relations in this work as “complex systems of personal and social relations through which women and men are socially created and maintained and through which they gain access to, or are allocated status, power and material resources within society” (Barriteau, 2001, p. 26). A central element of the social construction of the gender order is that a hierarchical difference is deeply inscribed into this relation, awarding men the more active and dominant positions and women the passive and subordinate status. Furthermore, this order is continuously reproduced by men and women themselves acting and thinking along these differences (West and Fenstermaker, 1996). However, such a bipolar gender order must be seen as part of more complex inequality relations, which are hierarchical in principle but become all the more complex when other social categories (such as class, race, and heteronormativity) are added (Fenstermaker and West, 2001).

### Literature Review

Sports-related research long focused on gender or race but not on the intersections of these two categories. This has changed somewhat, and there is a growing recognition that it is important to understand how gender and race interact to address prejudices and inequalities of opportunity in sports. Sutherland (2017) analyzes the intersections of race and gender in sports and the related interwoven discourses on racial stereotypes and gender labeling in a review article and shows that these have played an important role for a long time. Drawing on historical developments, the author illustrates that sexist and racist ideals in sports have existed for centuries and that they still determine how certain people are thought and spoken about and what position they can achieve in the world of sports. It becomes apparent that, historically, sports has always been a context where men and white individuals have the most power and which excludes (non-privileged) women and non-white men or makes their advancement more difficult (Sutherland, 2017). Empirically, this is shown by the fact that girls and women from ethnically and racially marginalized groups are underrepresented as sports participants and as professional sports leaders (coaches, administrators) (for an overview, see Carter-Francique and Flowers, 2013). Similar results were found with regard to the portrayal of marginalized sportswomen in the media, which was, at the same time, characterized by stereotypical and racist images of women (Carter-Francique and Flowers, 2013). Within this general corpus of research on intersections between race and gender in sports, there are also studies that deal with the specific setting of PE, in which the link to out-of-school sports contexts is established and which is framed by the general structures and logics of the extracurricular sports system.

Using intersectionality as an analytic tool in PE research is relatively new (Stride, 2016). The studies by Benn from the 1990s onwards are regarded as the first in the area of PE that take overlaps in categories such as gender and race into consideration (Scraton, 2001). Today, many researchers agree that race and gender are inextricably linked and that it is important to consider the intersectional perspective in order to understand how opportunities for participation in PE are structured (Dagkas and Hunter, 2015). Various studies based on the intersectionality approach have shown how current power relations in PE are framed by Eurocentric thinking and how this leads to the exclusion of certain groups of young people on the basis of their gender and their religious, ethnic, and/or cultural backgrounds (Flintoff, 2015; Azzarito, 2016; van Doodewaard and Knoppers, 2016; Thorjussen and Sisjord, 2018, 2020). This goes hand in hand with the findings on out-of-school sports and indicates that the privileged treatment of certain groups in out-of-school sports is thus already initiated or at least consolidated in PE. More specifically, the studies have shown that many teachers attribute specific behaviors to girls and boys with a migrant background in PE and that these young people are often confronted with stereotyped patterns of expectations that are structured along gender differences and racial lines. Muslim girls in particular, on whom research to date has focused, are often seen as inactive, unskilled, and disinterested in PE (e.g., Benn et al., 2011; Dagkas et al., 2011; Benn and Pfister, 2013). Non-Western immigrant boys, however, are also affected by stereotypes and often stigmatized as the “subordinate other” through discourses of hegemonic masculinity. They are often perceived as needing to be disciplined on the basis of supposedly Western norms of justice and order, such as “hard work, puctuality, […] humility, cleanliness and Christian […] cultural supremacy” (van Doodewaard and Knoppers, 2016, p. 13). Research has brought to light that PE teachers tend to view students who do not fully meet these normative white and Western ideals in PE through a deficit paradigm and regard PE as a place to “show them the way” (Barker, 2019, p. 141). Students who do not fulfill these assimilative requirements are considered as “the ‘Other’ as ‘different,’ ‘lacking something,’ ‘deficient’ or at best, ‘exotic”’ (Azzarito et al., 2017, p. 636).

However, these studies have not specifically addressed the field of forced migration, which evokes particular chains of association and stereotypes. To date, only a few papers have been published that deal with learners from refugee backgrounds in PE and also take gender aspects into account. Uptin et al. (2013) conducted a qualitative study with students from refugee backgrounds in Australian high schools. The researchers identified school sports as one of the few inclusive spaces in school, which enabled the boys from refugee backgrounds to present themselves as skilled athletes and to generate social capital. The girls, by contrast, did not perceive school sports as a way to make friends. They had to look for other ways to connect with peer groups, such as through music, which underlines that school sports limit participation opportunities for female students from refugee backgrounds. The results of an interview study with PE teachers by the authors of this paper on inclusion and the management of heterogeneity in German PE indicate that teachers perceive students from refugee backgrounds in PE in a stereotypical way and formulate gender-typical attributions along the hierarchical gender order particularly strongly (Bartsch et al., 2019). This study also showed that postcolonial patterns are reflected in PE by using it to teach obedience, convey Western Christian norms, and promote monolingual standards (Bartsch, accepted). However, these patterns need to be analyzed more closely and in a gender-sensitive way. A recent study shows that for some young women from refugee backgrounds at an Australian high school, school sports is a place where they have been able to develop a feeling of belonging, diminish cultural differences, and discover other facets of their identity (Harwood et al., 2020). However, the study goes on to describe how sports also cause tensions due to its gendered logics, patriarchal structures, and dominant values that conflict with those of the female students from refugee backgrounds (e.g., social and body practices). On the basis of Bourdieu, the researchers conclude that the habitus of female students from refugee backgrounds does not correspond to the habitus that would have made successful participation in school sports possible.

This research overview indicates that the analysis of intersections between race and gender in (school) sports is a fruitful approach when trying to come to a differentiated picture of power relations in sports. Yet, theoretically inspired and at the same time empirical works dealing with the relationship of *forced migration and gender* in sports are scarce. In addition, Spaaij et al. (2019) concluded in a review that the theoretical approaches of intersectionality and postcolonialism have been neglected in the field of sports and forced migration. These research needs will be addressed in this article.

### Theoretical Perspectives

Against the background of the points outlined above, we draw on the theoretical perspectives of constructivism, intersectionality, and postcolonialism in order to gain a more comprehensive understanding of the relationship of forced migration and gender in sports in general and teachers' perceptions of students from refugee backgrounds in PE in particular. A fundamental assumption of social constructivism is that categories such as gender, race, and social class are products of social attributions and thus social constructions (Carter-Francique and Flowers, 2013). Within the framework of constructivist analyses, reference is now increasingly being made to the interaction, interdependence or intersection of various categories of difference. Intersectional approaches, which emerged in the 1960s and 70s from the work of black feminists whose particular situation due to racial discrimination was hardly recognized or appreciated, focus on such intersections and the multiple oppressions associated with them (Hill Collins and Bilge, 2016). According to Davis (2008), intersectionality is defined as “the interaction between gender, race, and other categories of difference in individual lives, social practices, institutional arrangements, and cultural ideologies and the outcomes of these interactions in terms of power” (p. 68). The intersectional approach overcomes studies focusing on bipolar power relations that are not able to cover or detect the necessary complexity of social inequalities in society. Thus, the concept helps to foster deep analyses of social realities and sheds additional light on those marginalized groups (e.g., black women, lower class women, homosexual men, etc.) who are often neglected when doing one-dimensional studies that only take into account one category of social inequality (e.g., gender, migration status, class) (Crenshaw, 1989; Davis, 2008).

Yet, feminist theorists have particularly claimed that the complexity and open-endedness of intersectionality lead to ambiguities that might raise difficulties in concretely applying it to studies. While the application of intersectional approaches to empirical research certainly still involves uncertainties and vagueness, Davis (2008) concludes that, paradoxically, the concept's very lack of precision and its missing pieces are what have made it a successful approach and a useful heuristic tool for critical feminist theory. Furthermore, it is one of the concept's greatest advantages that it places extra emphasis on the specific context and setting in which power relations develop. As the name suggests, the guiding principle of this approach is the idea of intersections, where multiple categories of difference come together and create a unique pattern of social inequality that varies from context to context (Crenshaw, 1989). From this angle, the interaction of categories such as race and gender does not automatically lead to a mere addition of inequalities or to a double burden. Rather, it is assumed that the meaning of each category varies within different contexts and spaces, without there being a generally predetermined pattern between categories (Crenshaw, 1989). Following these implications, it is important for research in the field of intersectionality to focus on the contextualization of intersections. Consequently, the setting of sports and PE needs to be described for this work. The system of sports is a body-centered system that—looking back in its history—has predominantly developed as a field for boys and middle- or upper-class white men (Pfister, 2010). It is still a system in which (white) men are privileged and in which traditional gender roles and stereotypes are deeply rooted (Cunningham and Sagas, 2008). Furthermore, it is closely related to specific Western discourses of competition and success orientation and thus fosters multiple possibilities for power relations of inclusion and exclusion, domination and subordination. PE is the only body-related subject at school and is also linked to special learning locations (sports hall, swimming pool), particular methodological-didactic possibilities, and a strong orientation toward the extracurricular competitive sports system. However, PE not only reflects the structures and discourses of extracurricular sports but is also part of the educational system. It is therefore also about fulfilling the educational mandate, which states that all children and young people should have equal opportunities regardless of their origin, gender, and economic situation (UNICEF, 1989). PE can thus be described as a dynamic space in which different system logics and expectations converge.

The postcolonial perspective “can be seen as meaningful in a complex and multidimensional field of research, which can be understood as an interface of the discourses of ethnicity, race, gender, identity, and nationality within the discourse of globalization” (Günter, 2016, p. 629). It is inspired in particular by the publications of Said, Spivak, and Bhabha and assumes that colonialism, with its crimes and the beliefs on which it is based, has not yet been overcome despite liberation from colonial rule, so that society still must grapple with the effects of colonization in the present (Ashcroft et al., 2000). Constructivist and intersectional ideas are central components of postcolonial theory formation. The concept of intersectionality is particularly taken up by scholars from the field of postcolonial feminism, who deal with the intersections of gender with other categories in the context of postcolonialism and draw attention to the fact that women were especially instrumentalized during colonial rule. Patriarchal practices in some colonies were seen as evidence of the backwardness of colonized societies, and the liberation of women was therefore identified by the colonial powers as a crucial task that legitimized the establishment of asymmetric power structures (Castro Varela and Dhawan, 2009). This claim to the liberation and emancipation of women from the hands of their men by the colonial powers is symbolized by the phrase “white men save brown women from brown men,” which Spivak (1988, p. 296) coined in her key work of postcolonial feminist theory, *Can the Subaltern Speak?*. The construction of the Europeans as civilized rescuers and the “others” as underdeveloped, which is expressed in this sentence, enabled the colonial rulers to strengthen themselves and to establish and stabilize a discourse that constituted power over the colonized societies. This discourse and thus also the racialized and gendered construction of “we,” the West, and “the others,” the non-Western countries, has remained to this day. Even today, there are still one-dimensional and narrowing concepts that generalize non-Western women as oppressed victims and submissive recipients of male-dominated systems and make them appear as the antithesis of the supposedly progressive European woman (Samie, 2018). Equally dominant are depictions that identify non-Western men primarily as perpetrators and threatening, thus positioning them as opponents of the progressive and emancipated white man. However, these assumptions of non-Western women as a weak group to be liberated and non-Western men as an inferior group are not only taken up in postcolonial theories, but also in other feminist approaches. Of special note here is the concept of “hegemonic masculinity” developed by Connell, which has been used in gender studies since the 1980s to better understand the power of men over women and the dominance of men over other (mostly ethnically marginalized) men (Connell, 2015). The unique quality of postcolonial theories, however, is that they attempt to go beyond this and try to uncover and explain how the gendered and racialized structures and discourses affect non-Western and non-white individuals in the postcolonial world. Therefore, this work is also concerned with investigating the extent to which post-colonial discourses of othering are reflected in PE. By “othering,” we mean:

“treating difference between people hierarchically, for example, in terms of superiority and inferiority, thereby dismissing the needs of others as invisible or unimportant. The other not only functions as a way to maintain the interlocking systems of race, class and gender, but also as a way to reproduce a social, moral order in which people are positioned at the margins; the difference of the marginalized other maintains the mainstreamed center, the normal” (Dagkas and Hunter, 2015, p. 548).

These processes of othering and their underlying perceptions and attitudes can already be identified in colonialism's use of sports and PE as instruments of cultural imperialism and assimilation (Chepyator-Thomson, 2014). Sports were specifically used to shape and improve the character of those living in the colonies, by raising supposed Western virtues (e.g., punctuality, loyalty, self-sacrifice, obedience) denied to the colonized individuals, as the standard (Watson and Parker, 2014). The basic idea that the cultivation or disciplining of the “others” in sports can be advanced in a special way and more than in other areas has not lost its appeal even today. Thus, research studies still attest to a colonizing and formative character in contemporary sports and PE, which is closely linked to assimilation expectations of non-Western individuals (Hastie et al., 2006; Azzarito, 2016; Günter, 2016; Dowling and Flintoff, 2018). Assimilation means the adaptation of foreigners to the dominant norms and values of the host society, which demands the removal of all cultural differences. For Germany, however, a closer examination of PE from this perspective is still pending.

### Research Questions

Based on the current status of research and the theoretical outline provided so far, this article aims to analyze the intersections of forced migration and gender in one of the most basic and powerful organizational settings of sports, namely PE at schools. Against the background of constructivist, intersectional, and postcolonial approaches, we seek to analyze if and to what extent teachers' perceptions of students from refugee backgrounds are shaped by processes of “othering” and whether gender-related patterns are incorporated into these processes. In order to systematically analyze PE teachers' perspectives on students from refugee backgrounds, we focus on the teachers' perceptions and how they talk and express thoughts about these students. Specifically, the following research questions are examined:

Which interwoven gendered and racialized patterns of perception and interpretation are expressed when PE teachers talk about students from refugee backgrounds? How are these patterns shaped and how can they be interpreted against the background of constructivist, intersectional, and postcolonial approaches?

## Research Methods

The data presented in this article is based on a qualitative interview study with 31 PE teachers conducted between February and July 2017 in the federal state of North Rhine-Westphalia in Germany. The analysis carried out here is intended to deepen, broaden, and link the findings of two previous articles (Bartsch, accepted; Bartsch et al., 2019). The study was part of the overarching research project “Schulsport2020” [School Sport 2020], which aims to support both prospective and current PE teachers in dealing with heterogeneous learning groups and to promote equal participation in PE. A major focus of the interviews was to explore how teachers construct differences and how they deal with heterogeneous groups of students in PE. In this context, various social categories that influence chances of participation were examined (e.g., gender, disability, socioeconomic status). Due to social developments in Germany and the increased number of young people from refugee backgrounds, particular emphasis was placed on the issue of forced migration. The findings presented here represent an excerpt from this study and focus on PE teachers' perceptions toward students from refugee backgrounds. The interviewees were selected based on certain criteria. In order to gain basic insight into the subject area across all types of schools, the following aspects were taken into account: an approximate equal distribution of gender and membership of different types of school (representing the German school-system of Grund-, Haupt-, Real-, Sekundar-, Gesamt-, Berufsschule, Gymnasium)^1^. In addition, importance was attached to the fact that the interviewees had experience in dealing with students from refugee backgrounds^2^. Thirteen of the interviewees have practical teaching experience in so-called “Internationale Förderklassen” [international remedial classes], in which students from immigrant or refugee backgrounds are taught in a separate system until their language skills allow them to attend regular classes. The other interviewees have teaching experience in regular classes, in which all students are taught together. Some of the PE teachers were recruited via e-mail correspondence with schools, other via personal contacts. Of those surveyed, 18 are male and 13 female, and their average age is around 40 years (27–58 years). All have German citizenship and either profess the Christian faith or feel that they do not belong to any religion. With the exception of two interviewees who were born abroad (one in the USA and the other in Romania), all were born in Germany.

The data material was generated through problem-centered interviews, which, due to their semi-structured form and the possibility of more in-depth questioning, can stimulate richly substantiated narratives (Witzel, 2000). The average length of the interviews was 45 min (minimum of 20 min and maximum of 76 min). The interviews began with narrative-generating questions about the sports biography of the interviewees, their understanding of good PE, and their interpretation of the terms “heterogeneity” and “inclusion.” The teachers were then asked an open question about potential and relevant differences between students in PE. Building on this, the interviewers asked the teachers about different social categories with regard to their significance for participation in PE, including the topic of flight and forced migration. This specific question aimed to determine individual perceptions of the participants and their processes of “othering” toward students from refugee backgrounds, which may also be present collectively or institutionally. The interviewees were asked the following key questions: From your perspective, do refugee backgrounds and the student living conditions associated with it play a role in PE lessons? If so, to what extent? What opportunities and challenges arise in PE lessons with students from refugee backgrounds? How is PE in which students from refugee backgrounds participate organized?

Since postcolonial approaches are methodologically oriented toward discourse analysis (Diaz-Bone et al., 2007), a procedure based on this was used to prepare the data material for this article. For this purpose, the transcribed interviews were first coded along the content that was thematized in the catalog of interview questions. The text passages of all interviews about students from refugee backgrounds were subsequently evaluated and interpreted with a discourse-analytical focus based on Jäger (2003). Jäger's (2003) analytical tool was used, as she had already tested it in an interview study to depict intersections of migration and gender. In accordance with Jäger (2003), the analysis was carried out in five consecutive steps: (1) outline of the current discourse on flight and historical classification; (2) generation of interview material and analysis of the content structure of the transcripts with regard to dominant figures of speech, attributions, and stereotypes (e.g., “we” — “the others,” “men as perpetrators” — “women as victims”); (3) synoptic analysis (comparison of the differences and similarities in the perceptions of teachers toward students from refugee backgrounds); (4) detailed analysis of striking text passages in consideration of symbols, metaphors, allusions; and (5) embedding of the findings in the discursive context and derivation of overarching statements. The main analytical work took place during the fourth step, which aimed at the disclosure of processes of “othering” and colonial patterns within the material and required a semantic examination of the transcript corpus.

## Findings and Discussion

In the following sections, the study results are discussed with regard to the research questions. In line with the research questions, we concentrate on those grids and main patterns in the material that allow us to tie in with the theoretical perspectives of constructivism, intersectionality, and postcolonialism. The presentation of the results is structured along four central patterns of perception and interpretation that follow gendered and racialized logics and are expressed when PE teachers talk about students from refugee backgrounds. These core patterns, which were identified in the course of the analysis, are: *victimization and vulnerabilization, notions of threat and impulsivity, claims for assimilation and normalization*, and *demands for discipline*.

### Victimization and Vulnerabilization

The analytical screening of the interview material reveals that gendered and racialized notions of vulnerability form a dominant lens through which students from refuge backgrounds are perceived by PE teachers. More specifically, it appears that in the interviews, students from refugee backgrounds are described to a certain extent as victims and fragile subjects. These images center mainly on females from refugee backgrounds who are perceived as particularly vulnerable in PE lessons. The following quotation, in which a PE teacher associates a girl who has fled with sexualized violence and abuse, illustrates this observation.

“I was at swim class. There was a kid who refused to change, a girl. […]. And I signal the girl to change. I as a man, I signal an Arab girl to undress. Which of course is a huge problem. […]. What do I know about the child? Has she been raped? Has she experienced death and who knows what else?” (RS2m, 28)^3^

In this interview excerpt, the PE teacher clearly expresses a high sensitivity toward the gender constellations in swimming and gender-related reasons for flight and threats in the context of war (e.g., rape, genital mutilation). With regard to the theoretical framing of the article, however, the quote also brings to light colonial perception patterns in which racialized women, in this case the Arab girl, are seen as particularly fragile and potentially suffering. Especially in this specific case, where the teacher has no precise knowledge of the girl's biography, such speculation-based attributions can reinforce unequal power relations and legitimize paternalistic practices in PE. Drawing on this quotation, it can further be assumed that swimming lessons, as a special form of PE characterized by a pronounced physical presence and a body-conscious dress code, can particularly actualize and strengthen these associations of suffering and vulnerability, which underlines the special role of sports in the perception of young people from refugee backgrounds. The analysis also shows that the perception of vulnerability, which affects females from refugee backgrounds in particular, is accompanied by thoughts that characterize this group as very needy and worthy of support and protection. This finding is consistent with colonial narratives in which the “other” women were often described as in urgent need of rescue from their “oppressive” cultures. The iconic phrase that “white men are saving brown women from brown men,” formulated by Spivak (1988, p. 296), symbolizes this way of thinking. At the same time, the abovementioned interview excerpt expresses a distancing from traditional colonial claims of the “white man” as the savior of “black woman,” since the teacher here at least expresses self-reflection and insecurity in dealing with the situation.

The colonial mindset, which ascribes a vulnerable position to females from refugee backgrounds from which they must be “rescued” by the heroic Western subject, is reflected in further parts of the interviews.

“Yes, the girls [from refugee backgrounds] come at the beginning and don't dare to do anything and are afraid. So that's clearly visible in all their behavior. And then I tell them, first of all, you don't have to be afraid here; nobody does anything to you here. And if he [male student from refugee background] does something to you, he'll have to deal with me. And then, when they realize that, they blossom. And it's so fantastic with a refugee class like this. We only have a few girls, but they notice they're really protected here. The boys [from the refugee class] can't freak out here like they want, shoot the balls back and forth and throw rackets around, and so on. And that's why ‘I’ [the girls] feel comfortable here and dare to develop myself and that works great.” (BK4m, 28)

At first glance, the concerned and protective attitude of the teacher expressed in this quotation seems to be understanding and sensitive in view of gender-typical reasons for women and girls to flee their home countries and the massive experiences of violence that specifically affect females from refugee backgrounds (Schouler-Ocak and Kurmeyer, 2017). However, it is precisely in this case, in which the PE teacher has no exact knowledge of the girls' past experiences and backgrounds, that this perception actualizes unequal power relations and disempowers the girls. Such presumptions contribute to females from refugee backgrounds being pushed into the role of the vulnerable victim in need of paternalistic protection, which complies with the concept of the “real” and “good refugee” (Szczepanik, 2016). This mindset of Western masculinist protection erases female agency and disqualifies girls and young women from refugee backgrounds from their own ability to act. At the same time, in the quote, boys from refugee backgrounds are described by the teacher as chaotic and out of control. The reference to equipment (balls, rackets), which is essential for PE, makes the description of boys from refugee backgrounds as “freak[ing] out” even more meaningful. This narrative renders male vulnerability invisible and neglects that males from refugee backgrounds have also experienced violence and suffering. This perception of female students from refugee backgrounds as a weaker group and male students from refugee backgrounds as inferior men by a white male teacher ties in with the concept of “hegemonic masculinity,” which helps better explain how a particular group of men (white, Western-civilized, heterosexual) positions itself over others to assert dominance over marginalized groups such as women, gay men, and non-white men from non-industrialized countries (Connell, 2015). The observation that it is mainly male PE teachers who perceive students from refugee backgrounds along hegemonic patterns runs like a thread through all the interviews and becomes particularly visible with regard to female students. In addition to the characteristics already elaborated, such as vulnerable and in need of protection, they are described by (male) teachers as particularly fearful, weak, shy, and passive, which is expressed through the use of comparisons, superlatives, reinforcing adverbs, and word repetitions.

“Yes, what struck me was that especially with the refugee girls, so from the ninth grade […]. Yes, they were *very* reserved with their bodies […]. They were *really very, very* withdrawn. […]. They were *very, very* reserved.” (GY6m, 24)

“Especially the girls from the international remedial classes, they are *very* disinclined. As far as boys are concerned, they are *very* timid and sometimes *very* submissive.” (BK6m, 26)

Certainly, the teachers' impressions of females from refugee backgrounds, reflected in the quotations, are based on their subjective experiences in class; the descriptions refer to their personal perceptions and truths. However, from the perspective of the theories on which this article is based, these perceptions contain logics that largely correspond to the image of the subordinate “other” woman that emerged in colonialism and still shapes the Western debate on migrant women and the patriarchal gender order today. Overall, this predominant perception of students from refugee backgrounds as vulnerable victims, which is expressed in interviews mainly concerning older girls from refugee backgrounds in secondary schools, may contribute to a limited understanding of their multiple characteristics and life concepts. Other perspectives on young females from refugee backgrounds seem to be completely overlooked in the teachers' reports on their perceptions—at least, other perspectives are not expressed in the interviews. This result goes hand in hand with the out-of-school sports discourse in which Muslim women in sports are perceived as “different, strange, incompetent and out-of-place” (Samie, 2018, p. 35). These narrowed views “may have the effect of a self-fulfilling prophecy as both students and teachers do not make efforts to overcome and disprove these clichés” (Benn and Pfister, 2013, p. 568). If female students from refugee backgrounds identify with these clichés, this may have an impact beyond PE and discourage them from developing self-confident and strong personalities and engaging in responsible positions in a sports context (e.g., in club management, as trainers).

### Notions of Threat and Impulsivity

Another dominant gendered and racialized lens that we identified in the analysis of the interviews and that frames teachers' perceptions of students from refugee backgrounds in PE are notions of threat and impulsivity. This pattern of perception, which contrasts with the ideas of racialized female vulnerability, is particularly evident in relation to boys from refugee backgrounds. Unlike female students from refugee backgrounds in PE, perceptions toward male students from refugee backgrounds are hardly shaped by thoughts of vulnerability and the need for help. Rather, according to the data material, they are perceived as suspicious and wild as a result of the characteristics of racial masculinity attributed to them.

“The [male] newcomers are often super quick-tempered and very excited and loud, moody.” (GY9f, 80)

“Sometimes they [the boys from international remedial classes] are quick-tempered—for example, when it comes to breaking the rules and such—and the teacher may not have seen it directly. Then there is a lot of shouting and screaming, which you don't find to that extent in regular classes.” (GY8f, 82)

“In PE classes [with male students from refugee backgrounds]… yeah […], it's, you know […] a higher aggression. But it's not necessarily trauma related. But there's a clear increase in aggressiveness.” (GY7f, 31)

“But there are also boys [from refugee backgrounds] who are very dominant, but then mostly too dominant, because they cannot express it linguistically and so do it through physicality.” (RS2m, 86)

Here, too, the quotations reflect the PE teachers' subjective perceptions based on their personal impressions. These perceived realities are based on individual experiences but also on subjective expectations, belief systems, and unconsciously internalized images of groups of people that are culturally and historically shaped and influenced (Munhall, 2008). The perceptions of males from refugee backgrounds expressed in the interview excerpts reveal historically rooted and colonially influenced gender-specific ideas that disregard the possibility that male bodies are also weak and fragile in the face of war and have experienced suffering, trauma, and violence. While the vulnerability of females from refugee backgrounds is often the focus of attention in the interviews, the fact that humanitarian crises such as wars affect all genders in serious ways—including men and boys—tends to be neglected (Gray and Franck, 2019). The patterns of interpretation that shine through in the passages echo the colonial discourses of non-Western men as dominant and aggressive, which have gained specific impetus in Germany in the course of the narrations about the 2015/2016 Cologne New Year's Eve incident (Gray and Franck, 2019). These specific notions of boys from refugee backgrounds in PE, which relate primarily to older students, counteract the expectations placed on the “real” and “good refugee,” such as passivity or helplessness, which must be fulfilled in order to “deserve” protection. Persons who do not seem to meet the required normative characteristics of a “good” and therefore “ideal” refugee are more likely to be considered unworthy of protection and are sometimes even suspected of not having defended their country, of having left it for alleged reasons, or of abusing the social welfare system in the host country (Szczepanik, 2016). This mistrust of people from refugee backgrounds may make their settlement in the host country more difficult and may hinder their unprejudiced and unbiased positioning in the social subsystem of sports.

### Claims for Assimilation and Normalization

Demands for assimilation and normalization, in which closely interwoven racialized and gendered ideas are reflected and through which students from refugee backgrounds are perceived by PE teachers, emerge as a further key pattern in the interviews. In this context, the data show that teachers sometimes express assimilation expectations toward students who have fled, requiring them to adapt uncompromisingly to the social structures and the prevailing norms and values of the sports and school system in the host country. To emphasize the necessity of assimilation and to legitimize why they teach “more than sports” in PE classes with students from refugee backgrounds, the interviewees refer to ethnic deficit discourses (van Doodewaard and Knoppers, 2016), which are particularly shaped by ideas of oppressed female colonial subjects and their need for emancipation.

“A critical approach to the entrenched structures of Islam, such as the lack of equal rights for women. These are important things, and I believe that it is an important factor, especially when I have children [who have fled] from the Islamic culture, that women's equality is given priority. And this can of course be implemented well in sports. […]. I also find the headscarf very critical. I also think that it is still a symbol of oppression, and I also talk about it with parents. […]. I don't want a general ban, but I think that an influence on a cultural change is necessary.” (GY2m, 164–167)

In this quotation, the teacher generally articulates an increased awareness of gender-based oppression practices and patriarchal, hierarchical gender relations, which should be taken seriously as this can influence the participation of girls and women in school and sports. That the teacher wants to prompt “a cultural change” can be interpreted as well-intentioned, but at the same time, this statement reveals demands for assimilation connected with an inevitable adaptation to the prevailing Western Christian norms and values, which are still regarded as standard in Germany despite the undeniable development toward a migration society. When such perceptions are expressed collectively, this can lead to girls from refugee backgrounds generally being identified as problem cases in sports and PE, which neglects the diverse gender and role models in these young peoples' home countries. Furthermore, this image disregards the fact that the orientation toward cultural and religious traditions and familiar structures also has an identity-forming quality and can serve as a stabilizing resource in the unfamiliar reception context. Furthermore, such demands for normalization and assimilation alienate young people from refugee backgrounds from the awareness of their own identities and push them to seek new self-images and new consciousnesses, which can have problematic effects on their self-education processes and personality development (Azzarito, 2016).

In the opinion of some interviewees, these processes of assimilation and normalization can be implemented particularly well in the field of sports, especially because of its mostly coeducational structure and physical nature, which are used specifically to point out Western normalities to which young people should be introduced. Some teachers systematically use these opportunities to initiate assimilation and normalization processes that are intended to change young people from refugee backgrounds and free them from non-Western structures that are perceived as problematic.

“The girls [from refugee backgrounds], of course, as I had just explained, also have contact difficulties, especially now, when I do projects like this, like pyramids or acrobatics. But I do it extra natural. […]. It all makes sense why I do it. Not to play any games where they are far away from each other.” (RS2m, 25)

“We teach coeducational first. I think it is very important that exactly this […] strong separation of boys and girls, which the girls' society is doing, that we dissolve it completely. And also to use this normality that arises as an important social factor.” (GY2m, 82)

“We do it here as a normality that in sports you also touch each other, that you do it together, even physical confrontations. And I think that also shapes [the girls from refugee backgrounds]. That you take this image of society with you from sports.” (GY2m, 86)

These citations indicate that teachers work on the assimilation of their students through specifically selected forms of organization and movement, which in this way are only possible in PE and not in other school subjects, for example, by staging situations with focused physical contact. Assimilation efforts are thus latently reflected in pedagogical and educational demands that influence the teaching structure and the teachers' formation of PE lessons with learners from refugee backgrounds. These specific pedagogical ambitions are especially expressed by male teachers toward female students from refugee backgrounds, which once again reveals patterns of hegemonic masculinity (Connell, 2015).

### Demands for Discipline

The analysis of the interviews suggests another gendered and racialized pattern that develops when PE teachers talk about students from refugee backgrounds: demands for discipline. These demands are primarily addressed to students in international remedial classes, which are predominantly attended by boys and young men from refugee and migrant backgrounds who are in middle and later adolescence. These attempts at discipline become apparent in the interviews from the way the respondents organize teaching in these classes. They structure PE lessons particularly tightly and clearly regulated and tend to arrange them in a chiefly teacher-centered manner and instruction-oriented way.

“The things that stray away from the narrow leadership are more often avoided with respect to the IFK students [students from the international remedial class] because, as I have just indicated, they have a problem adhering to rules. […]. I see a greater danger with the IFK students, which means that the guidance is even a bit tighter.” (BK2m, 60)

“You have to set a very clear structure. And then somehow, when it finally works out, you can move on to more freedom. But as controlled as possible. As tightly as possible and also very clear rules and also very clear sanctions. That means immediately stopping the lesson or […] letting consequences follow. Because otherwise, yes, the students will blow up any lesson. And that means there must be a very clear structure and consequence behind it.” (GY7f, 31)

“I think it's very important for the students [from the international remedial class] to learn to follow instructions. That is, when an oral instruction is given, to translate it into action.” (BK4m, 26)

In using this teaching design, the interviewees seem to react to their experiences in international remedial classes and to serious challenges, such as communication problems, the disregard for rules, and the unbalanced gender composition, which are also brought up as topics in the interviews. From our theoretical perspectives, however, the teaching styles described and the underlying perception of males from refugee backgrounds as impetuous and wild arouse associations with sports during colonial rule, when they were instrumentalized to shape the bodies and characters of the colonized societies (Chepyator-Thomson, 2014). This colonial idea of controlling, cultivating, and disciplining the body of the “other” that lies outside the West through sports and PE can also be detected in the quotes above. Thus, the teaching styles outlined above can certainly be understood as the maintenance of hierarchical power structures at the interface of gender and race, in which the West dominates, and the “others” are kept in subordinate roles reserved for them in the Western world on account of their gender and ethnic origin. This finding goes hand in hand with the observation that some teachers make PE lessons in the international remedial classes particularly intensive and strenuous. An indication of this is that the focus there is predominantly on one-dimensional forms of movement and traditional Western and competitive content (e.g., fitness/strength training, endurance training, ball games).

“Then we always do a super hard warm-up training, alternating, the trainee teacher who […] has completed an additional training as a fitness coach. […]. Of course, he's well trained, and I used to play American football, so I am used to extreme warm-up programs.” (BK4m, 31)

“There we do power stations or performance-oriented […] or last week we made an indoor biathlon in groups, where altogether 40 rounds had to be run. And then you have to drop something, and if you don't hit, you have to do a penalty round, something like that. And […] in the group exercises, we do guided gymnastics and then simple games. Dodgeball, for example, is, I don't know, complex. […]. But something like that, where you have to drop a ball, where you have to sit down […]. Where you [can act] without body contact and, at the same time, give out a primal scream.” (BK1m, 43)

These strenuous physical activities, which are mentioned in the quotes, are seen by some respondents at other points in the interviews as quite positive in coping with trauma and other flight-related stress. The decision to design the lessons so exhaustive may therefore have been well-intentioned. From a postcolonial perspective, the contents of the lessons can be interpreted as an effort to discipline the bodies and movement practices of students from refugee backgrounds according to Western norms. The promotion of Western fitness ideals of well-trained bodies, which shines through especially in the first statement (BK4m, 31), can also be located in this direction.

## Conclusion

In this article, we investigated internalized notions and perceptions of gender and forced migration among PE teachers. More precisely, the aim of the study was to uncover interwoven gendered and racialized patterns of interpretation and to explore whether processes of “othering” are inscribed into teachers' images of young people from refugee backgrounds. To this end, interviews with PE teachers were analyzed on the basis of the constructivist, intersectional, and postcolonial approaches. In general, the analysis of the data shows that gendered and racialized perceptions seem to be inscribed in teachers' mindsets, “through which refugees come to be read *as* [a] risk or as *at* risk” (Gray and Franck, 2019, p. 278). In detail, the analytical look at the interviews reveals four dominant patterns that follow gendered and racialized logics through which students from refugee backgrounds are perceived. These core patterns are: *victimization and vulnerabilization, notions of threat and impulsivity, claims for assimilation and normalization*, and *demands for discipline*. *Victimization and vulnerabilization* can be identified in the data material, especially with regard to females from refugee backgrounds, who are perceived as particularly fragile and worthy of protection in accordance with colonial thinking about “other” women (Spivak, 1988). This perception corresponds with the idea of the “good refugee” (Szczepanik, 2016). On the basis of the quotes, it can be assumed that these notions of racialized vulnerability, formulated through gender, are reinforced by the physical references in PE. Such an underlying racialized and gendered understanding is also evident in the *notions of threat and impulsivity* that exist, particularly in relation to boys who have fled and who are more likely to be constructed as wild, rampant, and aggressive and thus fail to meet the expectations placed on them as genuine and suffering refugees (Szczepanik, 2016). These ideas of racialized masculinity are expressed in a pronounced way in PE and may be reinforced when boys from refugee backgrounds use play equipment such as rackets or balls, which seems to evoke specific threat associations. This perception obscures the potential vulnerability of all persons from refugee backgrounds (Gray and Franck, 2019). *Claims for assimilation and normalization* are expressed by the interviewees in particular toward females from refugee backgrounds, who are identified as problematic due to their cultural and religious backgrounds. Because of its coeducational and body-oriented arrangement, PE is considered particularly promising in freeing the girls from their structures that are marked as deficient and introducing them to the Western world. *Demands for discipline* expressed in the interviews relate primarily to male bodies, which, in line with colonial thinking, are sometimes considered as to be cultivated and disciplined. These demands are implemented in PE through a tight organizational structure and a high degree of physical intensity. Moreover, traits of hegemonic masculinity become apparent across all four patterns, as it is male teachers in particular who stage themselves in a powerful position over male and female students from refugee backgrounds (Connell, 2015). Overall, the analysis reveals that the teachers' perceptions of students from refugee backgrounds are rooted in a wider framework of gendered and racialized understandings that are not new but are transferred to colonial modernity (Gray and Franck, 2019).

Black feminist researchers have drawn attention to the fact that these intertwined gendered and racialized perceptions and the resulting actions can lead to far-reaching consequences, such as discrimination (Crenshaw, 1989; Davis, 2008). It is also assumed in the present study that the elaborated perceptions of learners from refugee backgrounds may have consequences. These can have a significant impact on the lives of young people from refugee backgrounds in the host country in general and on their participation in sports in particular. The teachers' internalized notions play out in how they think about interacting with students from refugee backgrounds in PE, framing the way these girls and boys are treated, encouraged, and discouraged from participating in school sports and in out-of-school sports, and taught how to perform as young women and men in today's Germany. In this context, studies have already shown that the classic stereotype of people from refugee backgrounds as helpless victims and the associated notions of dependence and humanitarian need, on one hand, create a positive attitude and support for their arrival (Bansak et al., 2016). On the other hand, after resettlement, these perceptions can erode the equal participation of people from refugee backgrounds and their capacities to integrate as fully accepted members in their new environments in the long term (Hynie, 2018). In the school context, studies on *stereotype threat* have shown that teachers' stereotyped expectation patterns toward students with migrant backgrounds may affect teachers' behavior and students' performance. Negative stereotypes can have an impact not only on the school performance of the young people concerned but also on their sense of belonging and self-esteem and their social life (Appel et al., 2015).

Based on this work, it is conceivable that the identified processes of “othering,” the racialized and gendered stereotypes experienced in PE, do not remain without consequences. This is particularly likely because PE is a place of outstanding importance for young people's sports biographies, and the experiences gained there are thus fundamentally formative and have a profound influence on further sports-related activities and careers. This is particularly the case for young people from refugee backgrounds, for whom PE is often the first and sometimes the only sports-related reference point in the host country. It can therefore be anticipated that stereotypes experienced in PE, which force young people from refugee backgrounds into certain roles, affect those young people's participation in extracurricular sports. Females who have been confronted with the stereotype of the victim and “woman to be liberated” might absorb this into their self-image and apply it in sports. Furthermore, girls who see themselves as victims probably have less ambition to take on positions of responsibility in sports (e.g., in club management, as trainers). Research has also shown that people tend to associate leadership positions, including the coaching profession, with men and “male” characteristics, such as strength, confidence, assertiveness, and success orientation (Cunningham and Sagas, 2008). Females from refugee backgrounds, who are labeled as weak and passive, are not associated with these “male” characteristics, which makes it even more difficult for them to take on leadership positions in sports, even if they want to. Even for males who have fled their home countries, based on our findings, it can be assumed that it is difficult for them to access positions of leadership in organized sports. Although they are generally not considered weak, they are often stigmatized as wild, unreasonable, and temperamental. These are qualities that do not appear to be compatible with leadership positions in sports, for which, for example, objectivity, diplomatic skill, and reasonable action are considered necessary. In all, these possible consequences underline that teachers' perspectives on learners from refugee backgrounds can have restrictive, discriminatory effects on those learners' sports-related biographies based on intersections of colonial and gendered constructions. PE teachers, who have usually been socialized in sports themselves or are still active in central positions of organized sports, should be aware of this great responsibility.

Our study aimed to examine teachers' perceptions of students from refugee backgrounds in PE from constructivist, intersectional, and postcolonial perspectives. Like the teachers we interviewed, we, as researchers, have moved into the field of tension between the construction and deconstruction of differences. Apparently, not only PE teachers but also scholars must constantly reflect on the assumptions underlying and shaping their reference systems. We are aware that our biographies as white, female academics with German as a first language may have influenced the research and our readings of the interviews. By discussing the citations and the analysis procedure within the team of authors and with colleagues, we have tried to become aware of our own position and reflect it in the interpretation of the data. Our study can therefore be seen as a contribution to a large and complex field of research that needs to be studied in greater depth in order to be better understood.

## Implications

To break internalized notions that prevent young people from refugee backgrounds from taking up equal positions in sports and society, it is necessary to sensitize (prospective) teachers to the fact that PE is a place where stereotypes are created or reinforced. We therefore agree with other researchers (Flintoff et al., 2008; Azzarito, 2009; van Doodewaard and Knoppers, 2016) who have long argued that PE teachers should be self-reflective and informed about intersecting discourses on gender and race in sports and their consequences. What is needed, therefore, is a professionalization of PE teachers in which topics such as intersectionality, racism, and the aftermath of colonialism in the present are addressed (Karakaşoğlu and Doğmuş, 2016; Mecheril, 2018). Discussing these issues can help teachers deal self-reflectively with their own perceptions of others and reflect on the social conditions for creating stereotyped, racist, and sexist images (e.g., via media representations). An essential task here is to recognize and consider the plurality of educational biographies in migration societies and, at the same time, to reflect on and avoid stereotypical and stigmatizing attributions (Karakaşoğlu and Doğmuş, 2016; Mecheril, 2018). However, (prospective) PE teachers in Germany have so far barely been sensitized to (power) positions and colonially based dominance relations. Topics such as postcolonialism and the processes of othering, which deal with foreignness, stereotypes, and their intersections, have so far barely been integrated into the training and further education of PE teachers. Racism-related topics in general have long been neglected and downplayed in Germany and probably in other countries as well (Fereidooni, 2019). However, the present study confirms that there is an urgent need to firmly establish these topics to promote intercultural competence among PE teachers. Recent events that have exposed structural or systemic racism in Western societies, such as the USA and Europe, based on white supremacy and Western ideas of superiority, also underline this urgency.

## Data Availability Statement

The datasets generated for this study are available on request to the corresponding author.

## Ethics Statement

The study involving human participants was reviewed and approved by the Ethics Committee of the German Sport University Cologne. The participants provided their written informed consent to participate in this study.

## Author Contributions

This paper is a joint work of both authors. BR is in charge of the project based on whi*c*h the publication was written and submitted the project application. FB wrote a first draft of the manuscript. BR gave feedback on it and made suggestions for focal points and additions. On this basis, FB revised the paper. BR carried out the final revision. All authors planned the article together.

## Conflict of Interest

The authors declare that the research was conducted in the absence of any commercial or financial relationships that could be construed as a potential conflict of interest.

## Footnotes

^1^In Germany, children first attend primary school (Grundschule) starting at around the age of six and normally for four years. At the end of primary school, the decision is made on how to continue their education, and they are assigned to different types of secondary school. These school types are organized in a performance-oriented structure, with paths leading to academic higher education or to technical and vocational training. The secondary school system is divided into Hauptschule (for less academically oriented students), Realschule (for intermediate-level students), Gymnasium (for academically oriented students). In addition to this hierarchical structure, there are also comprehensive and mixed secondary school types combining all education types (Gesamtschule, Sekundarschule), as well as vocational schools (Berufsschule).

^2^The schooling of newly immigrated students is not uniformly regulated in Germany; it differs, for example, in terms of the federal state, the educational stage, or the student's age. In the federal state of North Rhine-Westphalia, where our study was carried out, various schooling models for students from refugee backgrounds exist. Newly arrived students can be taught in specially established classes, so-called “Internationale Förderklassen” [international remedial classes], where they are educated separately until their language skills allow them to attend regular classes, as well as in regular classes (possibly with additive language support measures). In primary school, students are more often enrolled in regular or inclusive classes. In secondary school, especially vocational schools, it is more common for students from refugee backgrounds to attend separated classes that primarily focus on language development but may foster segregation.

^3^The notation of the interview quotes is composed as follows: (1) school type at which the interviewee works (GS, Grundschule; HS, Hauptschule; RS, Realschule; SEK, Sekundarschule; GY, Gymnasium; GE, Gesamtschule; BK, Berufsschule); (2) number assigned to the teacher out of all the teachers of the respective school type; (3) gender (f, female; m, male); (4) line number under which the citation can be found in the interview transcript.
